# Exosome-based crosstalk in glaucoma pathogenesis: a focus on oxidative stress and neuroinflammation

**DOI:** 10.3389/fimmu.2023.1202704

**Published:** 2023-07-17

**Authors:** Lixiang Wang, Xin Wei

**Affiliations:** ^1^ Department of Ophthalmology, West China Hospital, Sichuan University, Chengdu, China; ^2^ Department of Ophthalmology, ShangjinNanfu Hospital, Chengdu, China

**Keywords:** exosome, glaucoma, trabecular meshwork, neuroinflammation, microglia, oxidative stress

## Abstract

Exosomes are membrane-bound tiny particles that are released by all live cells that contain multiple signal molecules and extensively participate in numerous normal physical activities and pathologies. In glaucoma, the crucial role of exosome-based crosstalk has been primarily revealed in animal models and *ex vivo* cell studies in the recent decade. In the aqueous drainage system, exosomes derived from non-pigment ciliary epithelium act in an endocrine manner and specifically regulate the function of the trabecular meshwork to cope with persistent oxidative stress challenges. In the retina, a more complicated regulatory network among microglia, retinal neurons, retinal ganglial cells, retinal pigment epithelium, and other immune effector cells by exosomes are responsible for the elaborate modulation of tissue homeostasis under physical state and the widespread propagation of neuroinflammation and its consequent neurodegeneration in glaucoma pathogenesis. Accumulating evidence indicates that exosome-based crosstalk depends on numerous factors, including the specific cargos they carried (particularly micro RNA), concentration, size, and ionization potentials, which largely remain elusive. In this narrative review, we summarize the latest research focus of exosome-based crosstalk in glaucoma pathogenesis, the current research progress of exosome-based therapy for glaucoma and provide in-depth perspectives on its current research gap.

## 1 Introduction

Glaucoma is a multifactorial disease characterized by progressive damage to the retinal ganglial cells (RGCs) and consequent visual impairment (1). Many pathological processes, including oxidative stress, chronic neuroinflammation, mechanical damage with elevated intraocular pressure (IOP), mitochondrial dysfunction, impaired micro-circulation, and extracellular matrix remodeling have been found to contribute to its irreversible damage to the retina yet the associated mechanisms remain elusive (2–4). Due to the complexity, chronicity, and irreversibility of glaucoma, current medical and surgical treatment which is mainly based on IOP management fail to provide sufficient efficacy in a number of patients who progress to end-stage glaucoma and suffer from poor vision and chronic pain (5). In the recent decade, the focus of glaucoma research and management undergoes paradigm shifts from the previous focus on IOP-induced damage to prolonged neuroinflammation and neurodegeneration, with persistent efforts on regenerative therapy to reverse the damage of the retina (6–8). As discussed in detail in our previous reviews, there is accumulating evidence that the breakdown of the blood retinal barrier (BRB) and activation of immune effector cells, including microglia, T cells, plasma cells, macrophages, and glial cells participate in the entire process of glaucomatous neuroinflammation and precede the development of retinal damage (9, 10). However, how immune cells are synchronized and organized to induce neuroinflammation is poorly understood and remains the frontier of glaucoma research.

Conventionally, signal molecules, including cytokines, chemokines, growth factors, and hormones are found to be regulators of cell states and contribute to maintaining tissue homeostasis and their imbalance have been extensively studied in glaucoma pathogenesis. Recently, a new way of intercellular communication based on exosomes has been revealed to play a significant role in glaucoma (11–20). Exosomes are tiny membrane-bound nanovesicles with a size of 30 – 150 μm that are constitutively produced by all live cells (21). Exosomes contain several active biological materials, including proteins, messenger RNA (mRNA), micro RNA (miRNA), small interfering RNA (siRNA), and transcription factors that can regulate the function of recipient cells (22). Although exosomes are ubiquitous in the body, they demonstrate the selectivity of action which is achieved by the recognition and internalization through surface receptors (23, 24). Emerging evidence from animal studies and *ex vivo* cell experiments describes the central role of exosome-based crosstalk between trabecular meshwork (TM) and non-pigment ciliary epithelium (NPCE) in the modulation of aqueous drainage in reaction to oxidative stress and the crosstalk among microglia, retinal pigment epithelium (RPE), and retinal ganglial cells in the regulation of retinal inflammation and neurodegeneration (12, 13, 15, 25–27). In addition, exosome-based therapy has been primarily explored in animal models of glaucoma and shows promising results, despite numerous knowledge gaps to be explored (28–35). This review comprehensively summarizes the physical, pathological, and therapeutic role of exosomes associated with glaucoma, focusing on oxidative stress and retinal neuroinflammation.

## 2 Exosome-mediated crosstalk between NPCE and TM in the modulation of oxidative stress and extracellular matrix remodeling

The NPCE and TM are key components of the aqueous humor production and drainage system and play a vital role in the regulation of IOP. In patients with primary open-angle glaucoma (POAG), stiffened extracellular matrix builds up in the aqueous drainage system of the TM and Scheme’s canal, leading to their dysfunction (36). Exosomes as constitutive components in the aqueous humor have been found to play a vital role in the regulation of the homeostasis of the aqueous drainage system (37). The oriented outflow of the aqueous humor from NPCE to TM results in the basically one-way transmission of signals between the two tissues. Although theoretically exosomes can be secreted by all live cells, the binding of exosomes with receptor cells is relatively specific and exosomes originating from RPE have little effect on TM cells compared to NPCE-derived exosomes (14). The whole uptake process of NPCE-derived exosomes by TM starts with the recognition of specific surface receptors and ligands and involves the Dynamin 2-dependent route of transportation, which primarily depends on the endocytosis route for internalization (38). In the last decade, a number of *ex vivo* studies have explored the delicate crosstalk between NPCE and TM, which support the positive role of exosomes in maintaining the function of TM, especially under stress conditions (11–13, 15, 16, 39–42).

Intraocular tissues are consistently exposed to oxidative stress and rely on the antioxidant system to clear reactive oxygen species (43). Patients with chronic glaucoma present with dysfunction of mitochondria and deficiency of antioxidants, which results in the elevated production of reactive oxygen species in the aqueous humor and intraocular tissues (44). Higher levels of oxidative stress markers, such as malondialdehyde, are significantly enriched both in the aqueous humor and blood samples collected from glaucoma patients, particularly POAG, compared to healthy control (45). The chronic challenge of oxidative stress is detrimental to TM, which triggers an inflammatory cascade and activation of apoptosis and leads to the dysfunction and blockage of the aqueous drainage pathway (46). In addition, the antioxidant defense mechanisms of TM are compromised as the TM is not naturally exposed to the sunlight like cornea or iris, thus the TM is found to be particularly sensitive to oxidative damage (47). Exosomes produced by NPCE have been found to act as signal transmitters and break the vicious cycle of oxidative damage and TM dysfunction in glaucoma pathogenesis, as emerging evidence supports their role in the protection and modulation of TM metabolism, including improving their endurance under oxidative challenge and the ability to refresh the extracellular matrix (11–15). In the face of oxidative stress, exosomes from NPCE carry high levels of carbonylated proteins, which are end products of oxidized proteins and act as signal markers to evoke the activity of catalase and superoxide dismutase in TM cells (39). TM exposed to exosomes derived from hypoxia-challenged NPCE exhibits a significantly decreased level of oxidative stress due to the induction of major antioxidant genes (11). However, such activities are not observed when exosomes derived from non-stressed NPCE are used, suggesting their role in alerting target cells and improving their resistance under stressed conditions (39, 48).

**Figure 1:**
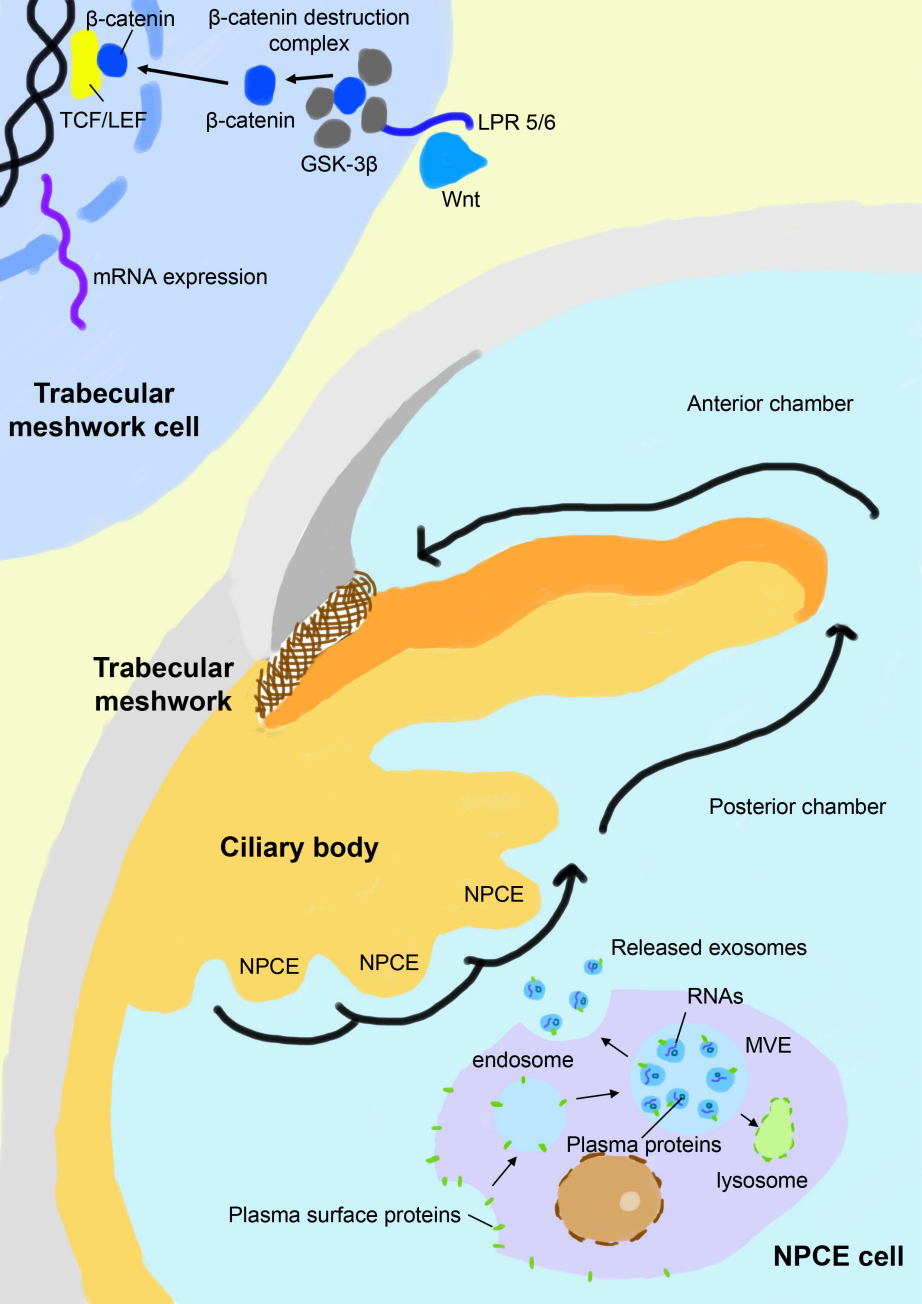
Schematic view of exosome-based crosstalk between NPCE and TM. In the NPCE cells, exosomes are constitutively formed from multivesicular bodies (MVE) after the inward budding of their membranes and are released with the fusion of MV with the plasma membrane. NPCE-derived exosomes specifically act on TM via oriented outflow of the aqueous humor to regulate its activities, particularly the canonical Wnt/β-catenin signaling pathway. Inhibition of the canonical Wnt/β-catenin signaling pathway in the TM is a signature of POAG, which is characterized by a reduced nuclear fraction of β-catenin and overexpression of its key negative regulator GSK-3β. Under the physical state, NPCE-derived exosomes positively regulate the Wnt/β-catenin signaling pathway in TM and promote the refreshing and phagocytosis of its extracellular matrix, which is compromised in patients with POAG. NPCE, non-pigment ciliary epithelium; GSK–3β, glycogen synthase kinase-3β; LPR 5/6, low-density lipoprotein receptor-related protein 5/6; TCF/LEF, T cell factor/lymphoid enhancer factor.

The chronic stress condition of the TM eventually results in its dysfunction in the modulation of the extracellular matrix and deposition of collagen fibers, leading to partial or complete blockage of the aqueous drainage pathway (49). The canonical Wnt/β-catenin signaling pathway is a key signal cascade downstream of the oxidative damage and is found to play extensive roles in many physiological and pathological processes, including cell proliferation, embryogenesis, inflammation, apoptosis, cell migration, and most relevantly, extracellular matrix remodeling and fibrogenesis (50–52). Activation of the canonical Wnt/β-catenin signaling pathway activates downstream genes involved in extracellular matrix recycling and refreshing and is found to antagonize dexamethasone-induced glucocorticoid receptor signaling in the TM (53). Inhibition of the canonical Wnt/β-catenin signaling pathway and overactivation of its key negative regulator glycogen synthase kinase-3β (GSK-3β) is a signature of glaucoma patients and is associated with elevated IOP (54, 55). In the canonical Wnt/β-catenin signaling pathway, the Wnt ligand binds with its receptor low-density lipoprotein receptor-related protein 5/6 (LPR 5/6) and disrupts the formation of the β-catenin destruction complex, leading to the enrichment of cytoplasmic β-catenin. β-catenin translocates to the nucleus, binds with the T cell factor/lymphoid enhancer factor (TCF/LEF) receptor, and activates related genes. In contrast, GSK-3β as a key component of the β-catenin destruction complex leads to hyperphosphorylation of β-catenin and subsequently activates its proteolysis (**Figure 1**). Exosomes released from NPCE have been found to elaborately modulate the canonical Wnt/β-catenin signaling pathway and related modulators, which seems to be both dose-dependent and time-dependent. Accumulating data suggests that NPCE-derived exosomes up-regulate the Wnt/β-catenin signaling pathway after their specific internalization by TM cells. A more than 2-fold decrease of the cytosolic fraction of β-catenin and conversely a significant increase of its nuclear fraction are observed in TM cells after exposure to NPCE-derived exosomes *in vivo*, accompanied by the decreased expression and phosphorylation of its key inhibitor GSK-3β (12, 13). In addition, a bimodal response of TM to NPCE-derived exosomes is revealed, which shows decreased level of β-catenin and its nuclear receptor LEF-1 when exposed to low concentration of exosomes compared to high concentration, suggesting the concentration of exosomes may play a key role in its differential regulation of TM metabolism (14). The modulation effects of exosomes are also found to be much more prominent in NPCE cell lines compared to NPCE primary cells, although they demonstrate a similar tendency to activate the Wnt/β-catenin signaling pathway in TM cells. Exosomes derived from NPCE cell line have a significantly larger volume and carries 5 times more protein content compared to primary cells (56). Thus, for therapeutic considerations, the selection of optimal cells for the collection of exosomes is vital to improve their therapeutic effects.

Dysregulation of extracellular matrix deposition is a hallmark of POAG, which increases the outflow resistance of the aqueous humor and leads to elevated IOP (57). Under physiological conditions, exosomes produced from TM and NPCE are found to participate in the opsonization and refreshing of its extracellular matrix, and this process is compromised in patients with POAG. Proteomic analysis reveals that exosomes derived from TM contain abundant proteins associated with extracellular matrix building and remodeling, including fibronectin, collagen, and integrin-binding ligand (15). TM secrete exosomes that assist the digestion of extracellular matrix components, including fibronectin and collagen type I (41, 42). For example, in a glucocorticoid-induced glaucoma model, excessive accumulation of fibronectin in the TM extracellular matrix results from reduced turnover by exosomes that bind significantly reduced amounts of fibronectin per exosome, which is consistent with a dramatic decrease of its binding proteins annexin A2 and A6 on its surface (58). Exosomes collected from non-glaucomatous individuals show a 55% increase in fibronectin-binding capacity compared to glaucoma patients, which can be further induced to improve by 63% after pre-conditioning with mechanical stretch, suggesting its regulatory role in reaction with stress conditions (15). In addition, exosomes secreted from NPCE demonstrate a positive role in the inhibition of TM extracellular matrix fibrosis by reducing the secretion of collagen type I, a key extracellular matrix component, by TM cells (40). Interestingly, TM exosomes from POAG patients are much smaller than healthy control due to the difference in membrane phospholipid content and phospholipid conversion enzymes, indicating decreased capacity to load extracellular matrix recycling proteins (59).

In addition to conditionally-expressed protein cargos, miRNA as another key component and regulatory factor of exosomes have been revealed to alter the gene expression of the TM and Schlemm’s canal, playing a significant role in glaucoma pathogenesis (16, 17). miRNAs are a group of non-coding RNAs that suppress the expression of their complementary mRNAs by cleavage, destabilization, or inhibition of their translation (60). As a core regulator in the post-translational process, a single miRNA may play multiple roles by interacting with numerous target mRNAs, and *vice versa*, which greatly diversifies the complicated regulatory network (61). As roughly estimated, more than 2,600 miRNAs have been identified in human since its first discovery in the 1990s (62, 63). Altered miRNA expression profile has been found to act as a signature of glaucoma in both experimental glaucoma models and patients (64–66). A panel of 20 circulating miRNAs have been identified to differentially express in glaucoma patients compared to healthy control, suggesting their role as potential biomarkers and pathogenic modifiers (67). Molecular analysis of exosomal cargos elucidates 584 micro RNAs (miRNAs) and 182 proteins associated with TM metabolism regulation (16). Accumulating evidence indicates that miRNAs as key cargos of exosomes regulate the response of TM to oxidative stress and alters the deposition of the extracellular matrix (16, 17). For example, tumor growth factor β2 (TGF-β2) is a key modulator of extracellular matrix remodeling and has been found to play significant roles in glaucoma pathogenesis (68, 69). TGF-β2-stimulated TM cells secreted exosomes that contain more than 2 folds the level of miR-7515 that promotes the expression of *VEGFA*, *VEGFR2*, *PECAM1*, and *Tie2* in Schlemm’s canal endothelial cells (17). After culturing with TGF-β2 for 24 hours, human TM cells produced exosomes that showed an upregulation of 23 miRNAs and downregulation of 3 miRNAs (70). Among these miRNAs identified, miR-29b as a major downstream effector of TGF-β2 has been most extensively studied, which is a suppressor of many extracellular matrix proteins of the TM (71, 72). By inhibiting the binding of nuclear factor-like 2 (Nrf2) to the promoter region of miR-29b, TGF-β2 suppresses its expression and promotes tissue fibrosis (73). In addition to its association with the TGF-β2 pathway, miR-29b is also a key modulator of the canonical Wnt/β-catenin signaling pathway, another important pathway involved in extracellular matrix remodeling as mentioned in the previous text (74). Exosomes produced from NPCEs carry abundant levels of miR-29b, and significantly suppress the expression of collagen 3A1 (COL3A1) in the TM cells *via* the Wnt/β-catenin signaling pathway (16). Thus, collective evidence suggests that exosomes produced by NPCEs may employ miRNAs as intracellular messengers to communicate with target cells and result in cell reprogramming.

Taken together, exosomes are constitutively released from NPCE and TM and serve a physical role in maintaining their homeostasis in oxidative stress response, apoptosis, and extracellular matrix remodeling. In patients with glaucoma, elevated oxidative stress and compromised function of exosomes form a vicious cycle that leads to increased resistance of aqueous humor outflow and elevation of IOP. Currently, researchers are aiming to stimulate and reacquire the normal function of TM with the help of external exosomes collected from mesenchymal stem cells, which is discussed in detail in part 4.

## 3 Role of microglia-derived exosomes in the spreading and modulation of glaucomatous neuroinflammation

For many years, RGC apoptosis by elevated intraocular pressure has been suggested to be the crucial etiology for irreversible visual impairment in glaucoma patients (75). However, in the recent decade, there is a paradigm shift that neuroinflammation induced primarily by microglia and T cells participates in the entire process of glaucomatous chronic neurodegeneration, which results in persistent and progressive damage to RGCs (9, 10). Microglia as constitutive housekeepers of neural tissues in the eye and the central nervous system are broadly distributed and serve many important physical functions in their quiescent form in maintaining the homeostasis of the neural tissue, including immune surveillance, control of neuronal excitability, organization of synapses, phagocytosis of cell debris, and secretion of trophic factors (76–78). When activated, microglia are highly heterogeneous and basically assume 2 different states in reaction to different physical conditions and stimuli, including the neurotoxic M1 microglia and neurotrophic M2 microglia (79, 80). Animal models of glaucoma reveal that microglia act as the earliest sensor and effector in glaucoma pathogenesis, which develop ahead of evident damage of RGCs (81–83). A pathogenic shift of microglial subtypes from M2-dominant to M1-dominant composition stimulated by pro-inflammatory markers such as interferon-γ (IFN-γ) is a hallmark of glaucoma neuroinflammation and is responsible for the synchronous activation of other immune effector cells, including cytotoxic T cells, plasma cells, and macrophages (see our previous reviews) (9, 10). Depletion of microglial activity by blockage of microglial adenosine A_2A_ receptor generates neuroprotective effects by suppressing the spread of neuroinflammation and subsequent RGC death in a rat glaucoma model (84). Collective evidence points to the central role of retinal microglia in glaucoma neurodegeneration.

**Figure 2:**
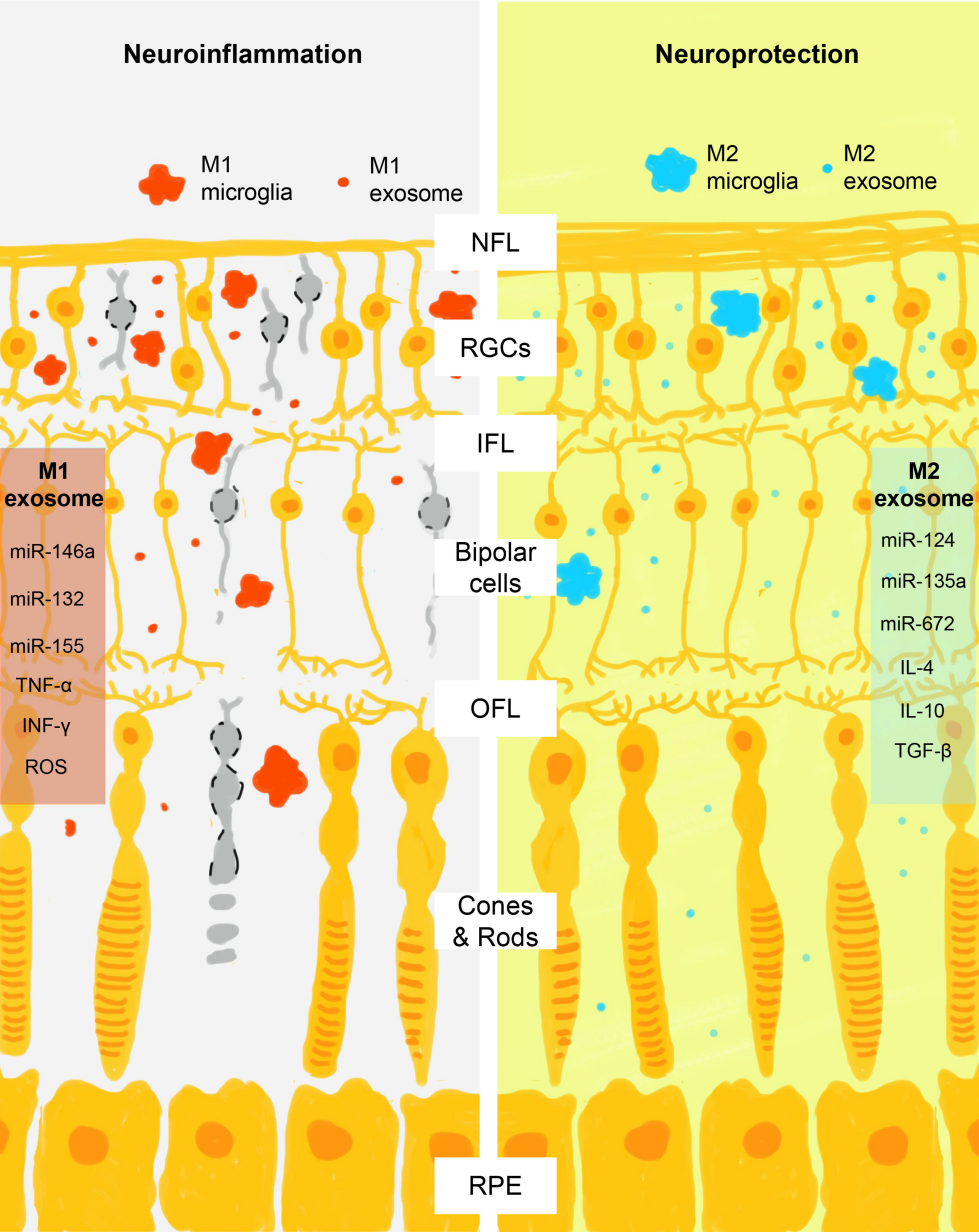
Schematic view of M1 and M2 microglia exosomes in the regulation of retinal neuroinflammation. After activation, microglia assume M1 or M2 phenotypes that act differently in the promotion and control of retinal neuroinflammation. Consistent with the action of M1 and M2 cells, their exosomes also contain phase-specific molecules, particularly miRNAs that regulate the activity of retinal neural cells through the complex crosstalk. M1 exosomes contain proinflammatory signals (miR-146a, miR-132, miR-155, tumor necrosis factor α, interferon γ, and reactive oxygen species) and M2 exosomes contain neuroprotective signals (miR-124, miR-135a, miR-672, interleukin 4, interleukin 10, and tumor growth factor β). NFL, nerve fiber layer; RGCs, retinal ganglial cells; IPL, inner plexiform layer; OPL, outer plexiform layer; RPE, retinal pigment epithelium.

Traditionally, microglia are supposed to spread neuroinflammation by physically wandering across the blood-retinal barrier (BRB) and secretion of corresponding cytokines and chemokines (tumor necrosis factor-α, interleukin-1β, superoxides, and reactive oxygen species for M1 microglia and interleukin-4, interleukin-10, interleukin-13, and tumor growth factor β for M2 microglia, respectively) (85, 86). Recently, researchers find microglia also employ exosomes to spread inflammatory signals in a paracrine and endocrine manner and serve as a key pathological component in many neurodegenerative diseases, including glaucoma, Alzheimer’s disease, and Parkinson disease (87–90). In the retina, the crosstalk between microglia, RGCs, and RPE is highly complicated yet elaborately organized due to the specificity of exosomes in signal transduction. Exosomes carry miRNAs and exchange them between origin and receptor cells to regulate their physical activities (91, 92). Studies find that microglia can amplify inflammation by selectively exchanging exosomes with different cell types, including microglia, RGC, RPE, retinal vascular endothelium, pericytes, oligodendrocytes, and astrocytes (25–27). In a rat retinal degeneration model, neural exosomes released from neural stem/progenitor cells that are injected into the subretinal space are found to be taken up primarily by microglial cells and result in immune modulation and protection of the retina (93). RPE is able to propagate signals of oxidative stress by sending exosomes from its apical site that contains damaged mitochondrial DNA, which are internalized by retinal microglia and induce a proinflammatory phenotype by activation of its cytoplasmic receptor Z-DNA-binding protein 1 (94). Conversely, microglia are able to regulate the production of exosomes in different states and modulate the immune background. Activated proinflammatory microglia not only multiply the production of exosomes but also load proinflammatory or neurotoxic molecules (95). Consistent with the subgroups of microglia, exosomes secreted by M1 and M2 microglia also contain distinct state-associated proteins and miRNAs that may participate in intercellular communication and regulation of recipient cells (96). For example, exosomes derived from M2 microglia exhibit neuroprotective ability on mice brain ischemic stroke model via exosomal miR-124 and its downstream target ubiquitin-specific protease 14 (97). On the other hand, M1 microglia-derived exosomes contain a distinct group of miRNAs, with the pro-inflammatory miR-146a-5p as their dominant miRNA component (98). As suggested by the current evidence, microglia seem to serve as an exchanging hub of exosomes with other cell types, and this bidirectional communication via exosomes leads to the change of their phenotypes and the spreading of neuroinflammation (**Figure 2**).

In glaucoma pathogenesis, accumulating evidence indicates that the activation of microglia is the prelude of neuroinflammation that rapidly respond to ocular hypertension challenge and assume a proinflammatory M1 phenotype within 24 hours (99–101). Emerging evidence suggests that microglia-derived exosomes may participate in the rapid spreading of inflammatory signals to other microglia and result in RGC degeneration (18–20). Microglia cells exposed to elevated hydrostatic pressure produced double amounts of exosomes that activate naïve microglia *in vivo* and improve their proliferation, phagocytic capacity, and mobility (18, 19). In addition, injection of such exosomes into healthy mice vitreous bodies induces propagation of intraocular inflammation and widespread RGC death (20). Compared with exosomes generated from naïve microglia, exosomes from activated microglia induced by elevated hydrostatic pressure contain increased levels of major histocompatibility complex II (MHC II), inducible NO synthase (iNOS), TNF, and IL-1β. Interestingly, such effects are generally abolished when GW4869, a neutral sphingomyelinase inhibitor that impairs the formation of exosomes, is added into the system, reinforcing the crucial role of exosomes in the spreading of neuroinflammation (20).

In addition to the pro-inflammatory effects of M1 microglia, M2 microglia may generate neuroprotective effects via their exosomes and are beneficial for immune modulation and protection of retinal cells. In a mice model of oxygen-induced retinopathy, microglia-derived exosomes injected into the vitreous body are selectively incorporated into photoreceptors and inhibit their apoptosis. These exosomes are found to contain high levels of miR-124-3p that inhibit hypoxia-induced activation of inositol-requiring enzyme 1α (IRE1α)-X-box binding protein 1 (XBP1) cascade in recipient cells (102). In addition, in the central nervous system, the protective effect of M2 microglia-derived exosomes has been extensively demonstrated in many different animal models, including Alzheimer’s disease (103), ischemic-reperfusion injury (97, 104), oxygen-glucose deprivation (92), and ischemic brain injury (105, 106). The neuroprotective effects are found to be basically induced by modulatory miRNAs carried in exosomes, such as miR-124, miR-137, miR-23a-5p, and miR-672 that target different singling pathways, including the Notch1, Olig3, and AIM2/ASC/caspase-1 pathways (97, 107–109). The activity of M2 microglia and their exosomes are vital to limit the intensity of neuroinflammation induced by M1 microglia and prevent extensive damage to the retina. However, in patients with glaucoma, chronic insult of elevated intraocular pressure, oxidative stress, excitotoxicity, and mitochondria dysfunction result in the imbalance of proinflammatory and anti-inflammatory microglia and their exosomes (110, 111). A shift of immune cells towards proinflammatory phenotypes is evidenced by autopsy studies (112–114) and peripheral blood samples collected from glaucoma patients (115, 116). As a result, researchers have proposed immunomodulatory therapy by applying regulatory exosomes, which are commonly collected from M2 microglia and stem cells, to shape microglia phenotypes (see part 4) (117).

## 4 Primary explorations of exosome-based therapy for glaucoma

Exosomes are crucial signal carriers that shape the state of recipient cells, and the imbalance of the production and malfunction of exosomes have been revealed to contribute to glaucoma pathogenesis both in the aqueous drainage system and the retina (16–20). In recent years, researchers have employed exosomes primarily originating from mesenchymal stem cells (MSCs) and induced pluripotent stem cells (iPSCs) to modulate the function of immune cells and achieve tissue regeneration in different animal models of glaucoma.

MSC-derived exosomes are currently the most extensively studied therapeutic exosomes in many medical fields, which have been promoted into clinical trials in patients with inflammatory bowel disease (118), chronic kidney disease (119), cerebral artery infarction (120), and demyelinating diseases (121). In the ocular system, exosomes as biological nano-sized particles have been primarily explored in several inflammatory and degenerative ocular disorders, including glaucoma, age-related macular degeneration, and anterior ischemic optic neuropathy (122–124). Compared with cell-based therapy, exosome-based therapy shows superiorities of great tissue compatibility, better penetration capacity, better immune tolerance, and the freedom to be modified with specific cargos to regulate target cells (125, 126). As with physical exosomes released by cells in the ocular system, external exosomes originating from MSCs are well recognized and free to distribute across the retina and taken up by microglia, RGCs, and retinal neural cells, resulting in the regulation of their activities (127). Exosomes show good retinal tropism after intravitreal injection and expand to diffused areas (128). When injected into the mice’s vitreous cavity, exosomes can efficiently reach the inner nuclear layer and outer plexiform layer, and to a lesser extent the outer nuclear layer as well (129). The rapid internalization of exosomes by the retina and microglia also attenuates their clearance and extends their effect time. Exosomes injected into the rat’s vitreous body remain detectable for as long as 4 weeks, with saturated binding to vitreous humor components (127). In addition, although MSCs are generally immune suppressive and show good tolerability, the immune-privileged intraocular tissues are highly sensitive, especially in diseased conditions, and the risk of inflammatory adverse events after MSC injection can’t be fully eliminated. As an example, intravitreal injection of 2 × 10^4^ bone marrow-derived MSCs and human adipose-derived stem cells into rat vitreous body elicits retinal glial activation, pericytes apoptosis, regression of retinal vessels, and formation of cataract with elevated levels of interleukin-1β, complex 3, arginase 1, and heat shock protein 90 (130). In a clinical trial, patients with retinitis pigmentosa receiving a single intravitreal bone marrow-derived MSCs showed mild adverse events, including mild macular edema, localized choroidal detachment, and the development of pre-retinal fibrous membrane (131). In comparison, injection of exosomes into the vitreous cavity is generally safe and no adverse events have been reported in animal studies so far.

As a cutting-edge research focus for glaucoma, exosome-based therapy demonstrates potential benefits in 3 basic aspects: modulation of neuroinflammation, protection of RGCs and retinal neurons, and promotion of tissue regeneration (28–35). However, currently, all evidence comes from *ex vivo* studies and animal models, with no clinical data available (summarized in **Table 1**). MSC-derived exosomes basically exert therapeutic effects via miRNA-dependent mechanisms, which is consistent with physical exosomes produced by intraocular tissues as discussed in the previous parts of the review. Consequently, the knockdown of Argonaute-2, a key miRNA effector molecule, diminishes the beneficial effect of intravitreal injection of exosomes produced by marrow-derived MSCs in a rat optic nerve crush model (33).The neuroprotective effect is also absent when fibroblast-derived exosomes are used, reinforcing the importance of cargo carried by exosomes (30). Interestingly, when original cells are primed in advance with appropriate conditions, the therapeutic effects of exosomes can be enhanced. For instance, in a mice chronic glaucoma model, the monthly injection of exosomes collected from tumor necrosis factor α-primed bone marrow-derived MSCs shows better efficacy of RGC protection compared with naïve exosomes (30). In glaucomatous rats, injection of exosomes from hypoxic MSCs but not normoxic MSCs exhibits beneficial effects by recruiting pro-regenerative macrophages into the TM and activation of ocular progenitor cells (28). The mechanisms of priming the original cells are still poorly understood, but researchers need to keep in mind the application of appropriate stimuli during cell culture to achieve better therapeutic outcomes. In addition, exosomes are excellent natural carriers of small and large molecules that show better penetration and distribution property compared to artificial nanocarriers (132). Modification of exosomes by changing their surface ligands, cargos, and insertion of specific genetic materials has become readily feasible and has been explored in different medical fields. For example, to enhance the activation of canonical Wnt/β-catenin signaling pathway in TM cells, NPCE-derived exosomes are genetically modified and loaded with SMAD7 small-interfering RNA (siRNA), which achieves a 53% knockdown of SMAD7 and elevation of β-catenin and tumor growth factor β2 in TM cells after incubation *in vivo (* 29). By conjugating Arg-Gly-Asp, a ligand for integrins that are highly expressed in choroidal neovascularization (CNV) tissues, exosomes derived from Müller glial cells or retina are able to selectively accumulate in areas of CNV after intravitreal injection and promote therapeutic effect (133). In considerations of glaucoma therapy, such surface modifications of MSC-derived exosomes may show potential benefits by enhancing their selectivity to target cells, such as microglia and RGCs, which need to be further explored and verified.

**Table 1:** Exosome-based therapy for TM remodeling and RGC protection in animal disease models.

Publications	Origin cells	Pre-conditioning of cells	Surface modification	Cargo modification		Animal model	Way and frequency of administration	Dose	Major outcomes	Adverse events
Exosomes targeting TM										
Tebid, 2022 (28)	MSC	Hypoxia	None	None		Laser-induced glaucoma, rat	N.A.	N.A.	Recruitment of macrophages for tissue regeneration	N.A.
Exosomes targeting the retina										
Mead, 2019 (30)	Human BMSC and iPSC	TNF-α priming	None	None		Chronic glaucoma model, DBA/2J mice	IVT, monthly	3x10^9^	↑RGC survival, ↓ axonal degeneration; TNF-α priming provides extra benefits	None
Mead, 2017 (33)	Human BMSC	None	None	None		Optic nerve crush, rat	IVT, weekly	3x10^9^	↑RGC survival, ↑ axonal regeneration	None
Yu, 2022 (31, 32)	GMSC	TNF-α priming	None	None		Retinal ischemia-reperfusion injury, mice	IVT, single dose	1 μg/2 μL	↓retinal inflammation, ↓RGC loss	None
Pan, 2019 (35)	UMSC	None	None	None		Optic nerve crush, rat	IVT, weekly	1 × 10^9^	↑RGC survival, ↑glial cell activation, no effects on axonal regeneration	None
Moisseiev, 2017 (34)	MSC	Hypoxia	None	None		Oxygen-induced retinopathy, mice	IVT, single dose	20 μg/1 μL	↑Vascular flow, ↑retinal thickness	None

TM, trabecular meshwork; MSC, mesenchymal stem cell; BMSC, bone marrow-derived mesenchymal stem cell; IVT, intravitreal injection; iPSC, induced pluripotent stem cell; TNF-α, tumor necrosis factor α; RGC, retinal ganglial cell; GMSC, gingival mesenchymal stem cell; ONHA, primary optic nerve head astrocyte; UMSC, umbilical cord mesenchymal stem cells.

In the field of ophthalmology, exosome-based therapy is just in its infancy phase of development. To promote the clinical translation of exosome-based therapy, great efforts have been made to lift the hurdles of manufacturing exosomes that comply with good manufacturing practices (GMP) (134). Major challenges come from the upstream standardized development, validation, and culture of cell lines and the downstream purification, collection, storage, and quality control of exosomes (135). Currently, only a few contract manufacturing organizations worldwide are available to provide clinical-grade exosomes under GMD regulation and no global consensus on the validation, production, and evaluation of exosome-based therapy is available, which limits the use of exosomes in clinical trials (136, 137). From the view of ophthalmologists, exosomes used for intraocular injection should adhere to a stricter standard due to the immune-privileged state of the eye in its physical state, despite their good safety profile demonstrated in previous animal models (138–140). Another important consideration for exosome-based therapy is its relatively short duration of action due to the clearance of exosomes and degradation of their cargos. This becomes a particular problem in glaucoma patients as glaucoma is a chronic and progressive disease and patients rely on long-term therapy to control IOP and protect their retina. In animal models, exosomes basically have to be injected into the vitreous body monthly or even weekly to achieve durable effects (30, 33, 35). However, the exact pharmacokinetics and efficacy of exosomes after intravitreal injection is poorly understood, especially for larger animal eyes such as nonhuman primates. Thus, the appropriate time gap between injections in patients is still unknown based on the current evidence. In addition, besides remarkable structural differences between animal eyes and human eyes, another gap between clinical translation and current animal models is the chronic pathogenic mechanisms of glaucoma that can’t be fully mimicked in acute phase animal models, such as optic nerve crush, acute elevation of IOP, and retinal ischemic injury models (141, 142). Whether frequent intravitreal injection of exosome-based therapy provides additional benefits over the conventional non-invasive application of eye drops and anti-glaucoma surgeries needs to be further evaluated. Moreover, a practice regimen that achieves RGC protection with lower injection frequency, potentially by combining the sustained release delivery system or using other routes of administration (suprachoroidal injection or subretinal injection) should be another focus of future research to improve patients’ compliance (143, 144).

## 5 Summary and conclusions

In this review, we thoroughly summarize recent research findings of exosomes in glaucoma pathogenesis and treatment. As constitutive nano-sized biological particles in the eye, exosomes participate in maintaining the homeostasis of TM and retina through complicated crosstalk between origin and recipient cells. The relatively free movement of exosomes within intraocular aqueous compartments and across BRB boundaries confers their capacity to rapidly spread signals in a paracrine and endocrine manner and shape the immune background of glaucomatous chronic neuroinflammation. As a potential therapy for glaucoma, exosomes originating from MSCs have been explored in multiple animal models and demonstrate neuroprotective effects. However, there are still obstacles between these emerging findings of exosomes in animal studies and clinical application, including knowledge gaps in pharmacokinetics, long-term efficacy, safety, and clinical-grade manufacturing. However, with boosting applications of exosomes in clinical trials in several other fields, there is great potential that exosome-based therapy may become readily available for clinical translation in the near future.

## Author contributions

Conception and design, LW and XW. Manuscript preparation, LW. Critical revisions, XW. All authors contributed to the article and approved the submitted version.
